# Feasibility and impact of a patient support group care model on diabetes and hypertension care in informal settlements in Nairobi, Kenya: a quasi-experimental study

**DOI:** 10.1080/16549716.2025.2482304

**Published:** 2025-04-09

**Authors:** Richard E. Sanya, Caroline H. Karugu, Samuel Iddi, Peter M. Kibe, Lilian Mburu, Lilian Mbau, Victor Kibe, Sloan Mahone, Naomi S. Levitt, Kerstin Klipstein-Grobusch, Gershim Asiki

**Affiliations:** a Chronic Diseases Management Unit, African Population and Health Research Center, Nairobi, Kenya; b Department of Public and Occupational Health, Amsterdam Public Health, University of Amsterdam Medical Centers, Amsterdam, The Netherlands; c Programmes, Kenya Cardiac Society, Nairobi, Kenya; d Department of Health, Nairobi City County Government, Nairobi, Kenya; e Faculty of History, Oxford University, Oxford, UK; f Chronic Disease Initiative for Africa, Department of Medicine, Faculty of Health Science, University of Cape Town, Cape Town, South Africa; g Julius Global Health, Department of Global Public Health and Bioethics, Julius Center for Health Sciences and Primary Care, University Medical Center Utrecht, Utrecht, The Netherlands; h Division of Epidemiology and Biostatistics, School of Public Health, Faculty of Health Sciences, University of the Witwatersrand, Johannesburg, South Africa; i Department of Women’s and Children’s Health, Karolinska Institutet, Stockholm, Sweden

**Keywords:** Hypertension, diabetes, self-financing, patient support groups, Africa, low- and middle-income countries

## Abstract

**Background:**

A support group care model including self-financing is a promising strategy to improve care for patients with diabetes or hypertension in resource-constrained settings.

**Objectives:**

We investigated the uptake, feasibility, and impact of a self-financing patient support group care model on cardiometabolic parameters among adult patients with uncontrolled diabetes or hypertension in informal settlements in Nairobi, Kenya.

**Methods:**

A two-group prospective quasi-experimental study was conducted. The outcomes were changes in mean glycated haemoglobin (HbA1c), systolic blood pressure (SBP), diastolic blood pressure (DBP), body mass index, and waist–hip ratio in control versus intervention communities, assessed 6 months after intervention implementation.

**Results:**

At baseline, 118 patients with diabetes (intervention, 60; control, 58) and 176 with hypertension (intervention, 87; control, 89) were enrolled. At endline, 81 patients with diabetes and 137 with hypertension were surveyed. In the intervention arm, HbA1c decreased from 10.8% to 9.0% (mean difference [95% CI]: −1.7 [−2.4, −0.9] *p* < 0.001) and in the control arm from 10.6% to 9.9% (−0.9 [−1.5, −0.3] *p* = 0.005). Difference-in-difference analysis showed a notably greater reduction in HbA1c in the intervention arm (−0.942 [0.463] *p* < 0.05). In the intervention arm, SBP decreased from 155.0 mmHg to 148.7 mmHg (−6.3 [−11.7, -0.9] *p* = 0.022) and in the control arm, from 160.1 mmHg to 152.5 mmHg (−7.6 [−12.9, −2.3] *p* = 0.005). DBP in the intervention arm changed from 99.1 mmHg to 97.9 mmHg (−1.1 [4.2, 1.9] *p* = 0.462) and in the control arm from 99.7 mmHg to 94.8 mmHg (−4.9 [7.8, −2.0] *p* = 0.001).

**Conclusions:**

A self-financing patient support group care model is feasible, improves cardiometabolic parameters and can be a strategy to manage diabetes, hypertension, and other chronic diseases in low-resource settings.

## PAPER CONTEXT

- **Main findings:** A co-created self-financing patient support group care model tested for 6 months among patients with uncontrolled diabetes or hypertension in informal settlements in Nairobi, Kenya, resulted in substantial reductions in glycated haemoglobin and blood pressure.
- **Added knowledge:** A self-financing patient support group care model for diabetes and hypertension is feasible and can impact disease outcomes in low-resource settings.
- **Global health impact for policy and action:** The self-financing patient support group care model is an additional toolkit to manage diabetes and hypertension in resource-limited settings and can be adapted for other chronic diseases.

## Background

Patient support groups have generated considerable attention as valuable tools in enhancing care for chronic illnesses [1]. These groups offer a platform for shared experiences, information exchange, and emotional support among individuals navigating these health challenges. They have shown benefit in the management of diabetes and hypertension [2–4], but most do not have a component of pooling financial resources. Self-financing patient support groups are self-help, peer-support groups set up by patients that pool group financial resources to support the purchase of medicines and/or self-care equipment. This model is expected to improve social cohesion, social support, and trust, and improve financial savings for accessing medications. A model of care centred around self-financing patient support groups is a promising strategy to improve care for patients with hypertension and diabetes in resource-constrained settings.

About 1.28 billion adults globally have hypertension, of whom two-thirds live in low- and middle-income countries (LMICs) [5]. In 2021, 537 million individuals worldwide had diabetes, a figure that is expected to rise to 783 million by 2045 [6]. In Kenya, 25% of the population has hypertension, and prevalence of diabetes is 2.4%, although likely to be an underestimate given that this number is based on fasting blood glucose and not the oral glucose tolerance test [7,8]. Public health facilities in LMICs, where the majority of populations access care, grapple with shortages in both workforce and medicine availability, leading to patients often bearing the brunt by paying out-of-pocket for medications. In Kenya, patients incur annual out-of-pocket expenses on medications ranging from $26 to $230 and $418 to $987 in public and private health facilities, respectively [9]. Only 24% of the Kenyan population had medical insurance coverage in 2022 [10], and people living in informal settlements in Nairobi are less likely to have medical insurance [11]. As public resources for managing patients with hypertension and diabetes are scarce in LMICs, including Kenya, innovative approaches need to be developed. Health initiatives that include microfinance offer opportunities to deliver services to populations in need [12]. Integrating microfinance and health interventions, such as health micro-insurance, linkage to health care, and access to health products, is beneficial [12–15].

There is paucity of data on whether self-financing patient support groups are feasible and effective in improving care among people with poorly controlled hypertension or diabetes. In this pilot work, we co-created a self-financing patient support group care model and investigated its uptake, feasibility, and impact, using a quasi-experimental approach, on cardiometabolic factors in informal settlements in Nairobi, Kenya.

## Methods

### Study design and setting

This was a two-group prospective quasi-experimental study of a patient support group care model versus standard of care conducted among patients with diabetes and/or hypertension in Viwandani and Korogocho informal settlements in Nairobi, Kenya, respectively. Korogocho and Viwandani are two informal settlements situated in Nairobi city, where the African Population and Health Research Center (APHRC) has maintained a Health and Demographic Surveillance System since 2002. Korogocho, positioned northeast of Nairobi and 12 km from the central business district, is the fourth largest informal (slum) settlement in Nairobi, covering approximately 1.5 square kilometres and hosting a population of 36,276 in 2018. Viwandani, closer to the central business district at 7 km, is located southeast of Nairobi, encompassing an area of 4–5 square kilometres and had a population of 52,698 in 2018 [16]. Both settlements exhibit relatively stable populations and are positioned 7 km apart from each other. The study was conducted between June 2022 and July 2023. We allocated the two communities to either the intervention or control (standard of care) through a lottery. Viwandani was drawn as the intervention community.

### Participants

This study included known patients with hypertension or diabetes who had the following inclusion criteria: adults (>18 years old) with uncontrolled blood pressure (systolic blood pressure ≥140 mmHg or diastolic blood pressure ≥90 mmHg) or glycaemia (HbA1c ≥ 7% and/or fasting blood glucose ≥ 7.2 mmol/L) at the time of screening, residing in the selected study communities with a commitment to be available for at least 6 months, willing to participate in the study and engage in peer support groups if part of the intervention community. Patients with suspected secondary hypertension according to local guidelines, those with difficulties obtaining valid blood pressure values (e.g. due to arrhythmias), pregnant individuals, and patients deemed unsuitable for participation due to life-threatening diseases, dementia, or recent acute cardiovascular events within the previous 3 months, were excluded.

### Intervention

**Figure 1.**
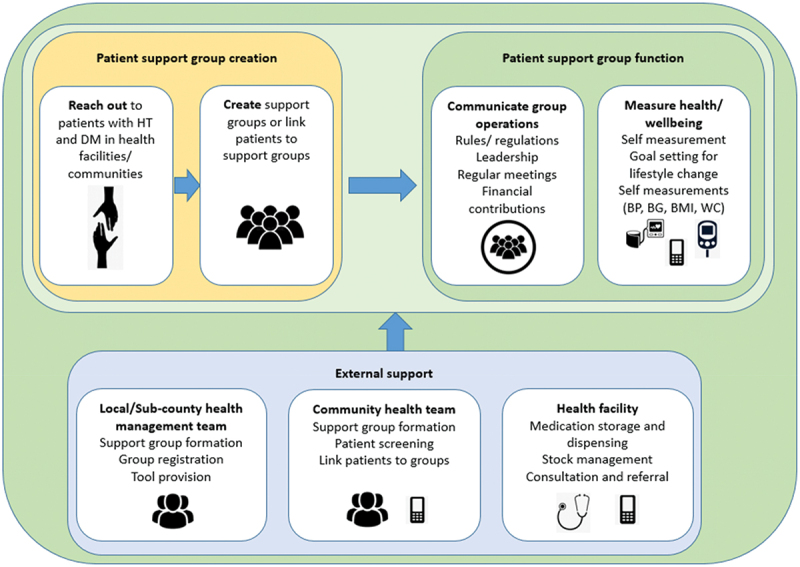
Self-financing patient support group care model for hypertension and diabetes.

A model of care for self-financing support groups for diabetes and hypertension was developed and refined (Figure 1) using a co-design approach. This framework of care is based on the longstanding experience of groups of patients with hypertension and diabetes who have been functioning independently with regular clinical support from a nurse from a local clinic (as observed by the authors). Following the UK Medical Research Council framework for developing and evaluating complex interventions, a systematic review [17] was conducted to establish the evidence base, focused interviews with patients and healthcare providers identified what components of the intervention might work; and key stakeholders were engaged to co-design the intervention.

The care model involves the creation and operationalisation of self-financing patient support groups for hypertension and diabetes. The groups receive external support from local health facilities, community health teams, and local government. The key components of the model include ‘reach out’ activities to invite patients with hypertension and diabetes to join the groups, group creation and membership, group function, group leadership, meetings, self-management tool provision, self-care, financial management, and support from external parties. The components are described in detail in Table 1.

**Table 1.** Self-financing patient support group model for hypertension and diabetes: major intervention components.

Component	Details
Reach out activities	Identify people in the community who are contacted by the study team. Develop the materials to explain and initiate the self-financing groups. Collect material and data to assess the success of the reach-out in creating a group.
Group creation and membership	Each group will have at least 10 members with no upper limit. To join a group, an individual should have a diagnosis of hypertension and/or diabetes and has expressed willingness. There will be no restriction based on residence, gender, or any other social grouping. Groups will be linked to public health facilities and registered. Community health volunteers and health workers will help in mobilising and encouraging patients to join the groups. Upon mobilisation of patients, group leaders will be identified. A representative from the health facility will be co-opted as a member of the support group and will train the group leaders. The groups will be registered with the ministry of labour and social services as self-help groups.
Group function	The groups will be governed based on rules set up by each group. This includes rules on membership, meetings, and finances.
Group leadership	Each group will have a chairperson, secretary, and treasurer. Groups can add other leadership positions depending on need and preference. Leaders will be elected by group members. Nomination of leaders will be by show of hands and voting will be by secret ballot. The groups will decide on term limits and the duration of tenure of the leaders. The leaders will guide/manage the groups based on rules set by the group. Leaders will be offered training based on need. This training can be from the health facility or offered externally.
Meetings	Groups will hold meetings with the frequency decided by the group. During these meetings, members will collect financial contributions, take measurements, receive health education, peer counselling, consultation, and social support. They can also discuss issues related to the welfare of the group. Each group will have an annual general meeting during which elections are held and major resolutions made.
Tool provision to group	Self-measurement tools such as sphygmomanometers, glucometers, and testing strips will be provided. Members will be trained on how to use these tools. The groups will give help with registers/recording tools to document meeting attendance and measurements. If available, educative materials on management of the conditions will be provided.
Self-care	In the groups, members will set goals for lifestyle change. During meetings, measurements of blood pressure and blood glucose can be taken. If equipment is available, weight, BMI, and waist circumference will be recorded. Records of patient measurements (e.g. blood pressure and blood glucose) and health status will be kept and shared with the health facility. Members will seek health education, peer counselling, and social support in their groups.
Financial management	Groups that agree to engage in financial activities will decide how much each member should contribute and how frequent the contributions should be. The contributions can be made in cash or by mobile money. Transparency and accountability are encouraged, and each group will come up with modalities for accountability. If required, training on financial management will be provided by the facility in-charge or from external sources.
Economic empowerment	The groups should not be saving credit schemes.
Health facility support	Groups will be linked to public health facilities (Level 2 and 3) to access care. The healthcare workers will help initiate the support groups. A clinician or nurse will support each group. Data flow between facilities and the groups will be encouraged. The health facility will support medication storage and dispensing, consultation, and referral. The health facility, if public/government-owned, will dispense the medications procured by the group only if there is a stock-out of government supplies of the said medications. The health facility team will also help in conflict resolution within the groups. Electronic data collection tools will be provided to the health workers (for one group).
Community health team support	Community health teams will assist in mobilising patients to join the groups and tracking group members.
Local government support	The sub-county health management team will assist with group formation, registration, and tool provision.
Other (external support to groups)	Groups can solicit external support (from family, relatives, friends, non-governmental organisations, pharma, government, and civil society) to supplement their activities.

### Implementation

A baseline survey was conducted, and eligible participants were recruited from the study communities through collaboration between the research team and community health volunteers. The model was rolled out in the intervention community. An inception meeting was held, and the model was explained to the study participants. The mobilisation process involved key stakeholders, including the health facility team, the Makadara sub-county non-communicable disease office, and the community health team. Group formation ensued, during which leaders were elected and rules were established. Participants were then equipped with blood pressure and blood glucose measurement devices and underwent training on their usage. The groups were linked to Lungalunga Health Centre. The group formation period extended from December 2022 to January 2023, resulting in the creation of three distinct groups: one with 50 members with diabetes, and two other groups comprising 44 and 43 members with hypertension only. The groups were followed up for 6 months, and an endline survey was conducted in both study communities in July 2023.

### Outcomes

The study’s primary outcomes were change in mean blood pressure and HbA1c levels, and the proportion of patients achieving controlled blood pressure, and HbA1c. Secondary outcomes included changes in body mass index (BMI) and waist–hip ratio. Implementation outcomes such as reach, effectiveness, adoption, implementation, and maintenance were also assessed.

Three systolic and diastolic blood pressure measurements were taken at 5-min intervals using a digital sphygmomanometer (OMRON Model M1 Basic [HEM-7121J-AF], Omron Health Care, Kyoto, Japan) with an appropriately sized cuff after participants had sat at rest for a minimum of 5 min. The average of the second and third measurements was used for the analysis. Controlled BP was defined as systolic blood pressure <140 mmHg and diastolic blood pressure <90 mmHg. HbA1c testing was done using the turbidimetric inhibition immunoassay method at the laboratories in Kenyatta University Teaching Referral and Research Hospital, Nairobi. Anthropometric measurements included body weight, measured with a mobile-use flat scale (SECA 874, Seca GmbH & co, Hamburg Germany) to the nearest 0.1 kg, height assessed with a portable stadiometer (SECA 213, Seca GmbH & co, Hamburg Germany), and the calculation of BMI using weight and height. Waist circumference was measured at the midpoint between the lowest rib and the iliac crest using a non-stretchable measuring tape, while hip circumference was measured at the level of the greater trochanters, with participants wearing light clothing. Behavioural aspects, encompassing smoking, alcohol use, diet, and physical activity, were assessed through a questionnaire adapted from the WHO STEPS instrument. Implementation outcomes, including reach, effectiveness, adoption, implementation, and maintenance, were systematically assessed, considering factors such as the percentage and type of patients reached. The intervention’s consistency, acceptability, and the sustained maintenance of intervention components and effects were assessed quantitatively by questionnaire and qualitatively through in-depth interviews with healthcare providers, patients who took up the intervention and patients who either dropped out or refused to take up the intervention.

### Sample size

Separate sample size estimates were computed for patients with hypertension and diabetes in the study. For patients with uncontrolled hypertension, the estimates were derived from a community-based study conducted by Van De Vijver et al. [18], showing that only 21.5% of individuals with hypertension in Nairobi slums receiving treatment, achieved controlled blood pressure levels below 140/90 mmHg. We assumed that the current intervention would increase BP control by 20% in 6-month follow-up (based on our unpublished findings from peer support groups attending clinics in Nairobi). Thus, we posited a 20% meaningful difference and 80% power with 5% level of significance (one-sided test) to detect that difference. Since our study design is a pre–post design, we estimated the sample size using the formula for paired proportions [19]. The minimum sample size required was 75 patients with uncontrolled hypertension per study arm. Assuming attrition of 20%, the final sample size was 90 per study arm.

For patients with uncontrolled diabetes, we estimated a sample size of 60 patients per study arm. In reference to a study by Otieno et al. [20], we estimated that among patients with diabetes who are in treatment, 30% achieve glycaemic control. We assumed that the current intervention would increase glycaemic control by 30% in 6 month follow-up based on findings from a randomised control trial in three LMICs including the Democratic Republic of Congo [21]. Thus, we posited a 30% meaningful difference and 80% power with 5% level of significance (one-sided test) to detect that difference. Since our study design was a pre–post design, we estimated the sample size of 49 patients with uncontrolled diabetes per study arm. Furthermore, we assumed a dropout rate of 20%, resulting in the final sample size of 58 (approximately 60) patients with diabetes to be enrolled in each study arm.

### Statistical methods

Statistical analyses were done using R version 4.2.2 (R Foundation for Statistical Computing). Descriptive characteristics were tabulated and presented as percentages and proportions for categorical variables and mean and standard deviations for continuous variables. The statistical analysis was by intention to treat. Paired tests (before and after) were conducted for outcomes in the control and intervention groups separately. Adjusting for independent factors, we compared the outcomes in the intervention versus control arm using mixed-effects linear models (difference-in-difference analysis) for continuous-type outcomes and generalised linear mixed models for the binary outcomes using random effects to account for repeated measurements. Thematic content analysis was used for qualitative data.

## Results

### Participants and intervention uptake

**Figure 2.**
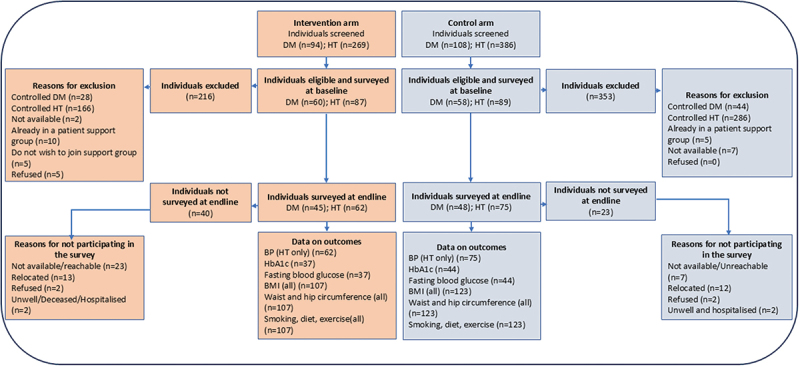
Flowchart of the quasi-experimental study to investigate the impact of the self-financing patient support group care model on individuals with uncontrolled hypertension or diabetes.

The flow of participants is shown in Figure 2. Overall, 202 patients with diabetes and 655 patients with hypertension were screened for eligibility. Of these, 118 patients with diabetes and 176 patients with hypertension were confirmed eligible and surveyed at baseline. After 6 months of follow-up, 81 participants (69%) with diabetes and 137 participants (78%) with hypertension were available for the endline survey (the dropout rate was higher than the 20% expected).

The characteristics of the participants are shown in Table 2. Most participants, 76% in the intervention arm and 73% in the control arm, were female. The average age of the participants in the intervention and control arms was 49 and 54 years, respectively. Most participants had formal education up to the completion of primary school and were mainly self-employed, running small businesses. Close to 20% were unemployed. The majority of participants had visited a health facility in the last 6 months to seek care for diabetes/hypertension, and about half of the participants reported that not all the medicines that they needed were available at the facility they sought care from. The most common reason for the unavailability of medicines was that the medicines were out of stock.

**Table 2.** Baseline socio-demographic and clinical characteristics of study participants enrolled in the quasi-experimental study to test the impact of a self-financing patient support group care model in informal settlements in Nairobi, Kenya.

Variable		Intervention (Viwandani) N = 147 N (%)	Control (Korogocho) N = 147 N %)	*P*-value*
**Socio-demographics**				
Sex	Female	112 (76.2)	107 (72.8)	0.514
	Male	35 (23.8)	40 (27.2)	
Age	Mean (SD)	49.0 (10.9)	53.9 (11.6)	<0.001
Education	No formal schooling	11 (7.9)	4 (2.8)	0.004
	Primary	90 (63.4)	92 (66.2)	
	Secondary	39 (27.5)	34 (24.5)	
	College/university	2 (1.4)	9 (6.3)	
Marital status	Never married	8 (5.6)	8 (5.8)	0.011
	Currently married/cohabiting	77 (54.2)	74 (53.2)	
	Divorced/separated	2 (1.4)	6 (4.3)	
	Widowed	19 (13.4)	34 (24.5)	
Occupation	Public servant	1 (0.7)	-	0.026
	Private formal sector	3 (2.1)	1 (0.7)	
	Self-employed (small/large business)	69 (48.6)	82 (59.0)	
	Homemaker	4 (2.8)	11 (7.9)	
	Casual labourer	26 (18.3)	21 (15.1)	
	Retired	-	1 (0.7)	
	Unemployed	36 (25.4)	17 (12.2)	
	Other	3 (2.1)	6 (4.3)	
Income (KES)	<1000	26 (18.3)	15 (10.8)	0.557
	1,000–2,499	24 (16.9)	27 (19.4)	
	2,500–4,999	25 (17.6)	24 (17.3)	
	5,000–7,499	21 (14.8)	26 (18.7)	
	7,500–9,999	12 (8.5)	8 (5.8)	
	10,000–14,999	19 (13.4)	23 (16.6)	
	15,000–20,000	11 (7.8)	14 (10.1)	
	>20,000	4 (2.8)	2 (1.4)	
**Alcohol and smoking**				
Currently smoking	Yes	5 (3.5)	10 (7.2)	0.171
Ever smoked	Yes	16 (11.3)	32 (23.0)	0.009
Frequency of alcohol use (last 12 months)	Daily	3 (2.1)	4 (2.9)	0.551
	Weekly	6 (4.2)	3 (2.2)	
	Monthly	6 (4.2)	3 (2.2)	
	Occasionally	9 (6.3)	13 (9.4)	
	Never	123 (86.6)	116 (83.5)	
**Diet and exercise**				
No. of servings of fruit in a typical week	Mean (SD)	2.9 (2.5)	2.9 (2.3)	0.926
No. of servings of vegetables in a typical week	Mean (SD)	6.3 (2.6)	7.0 (3.5)	0.059
How often salt, salty seasoning or salty sauces is added in cooking or preparing foods in your household	Always	129 (90.9)	74 (53.2)	<0.001
	Often	1 (0.7)	42 (30.2)	
	Sometimes	4 (2.8)	18 (13.0)	
	Rarely	2 (1.4)	1 (0.7)	
	Never	5 (3.5)	4 (2.9)	
	Don’t know	1 (0.7)	-	
How often salt, salty seasoning or salty sauces are added on food on the table	Always	12 (8.5)	6 (4.3)	<0.001
	Often	3 (2.1)	22 (15.8)	
	Sometimes	17 (12.0)	33 (23.7)	
	Rarely	22 (15.5)	12 (8.6)	
	Never	88 (62.0)	66 (47.5)	
	Don’t know	12 (8.5)	6 (4.3)	
How often processed foods high in salt are eaten	Always	12 (8.5)	1 (0.7)	0.005
	Often	3 (2.1)	8 (5.8)	
	Sometimes	23 (16.2)	29 (20.9)	
	Rarely	31 (21.8)	41 (29.5)	
	Never	73 (51.4)	60 (43.2)	
	Don’t know	12 (8.5)	1 (0.7)	
How often sugar is added to beverages right before drinking them or as they are drunk	Always	27 (19.0)	61 (43.9)	<0.001
	Often	9 (6.3)	23 (16.6)	
	Sometimes	15 (10.6)	16 (11.5)	
	Rarely	21 (14.8)	14 (10.1)	
	Never	69 (48.6)	25 (18.0)	
	Don’t know	1 (0.7)	0	
How often processed food high in sugar is eaten	Always	-	4 (2.8)	0.001
	Often	1 (0.7)	7 (5.0)	
	Sometimes	29 (20.9)	37 (26.1)	
	Rarely	49 (35.3)	25 (17.6)	
	Never	60 (43.2)	69 (48.6)	
	Don’t know	-	-	
Number of days of vigorous activity of ≥30 min in the past week	Mean (SD)	1.5 (2.3)	2.1 (2.4)	0.015
**Healthcare seeking**				
At any time did not get the needed care at clinic attended	Yes	63 (44.3)	87 (62.6)	0.002
	No	79 (55.6)	52 (37.4)	
Reason for not getting the care sought	There was no medication	58 (92.1)	73 (83.9)	0.138
	No healthcare provider	1 (1.6)	8 (9.2)	0.053
	Not able to pay	7 (11.1)	24 (27.6)	0.014
	No equipment for assessment	3 (4.8)	1 (1.2)	0.175
	Other	4 (2.8)	30 (21.6)	
In the last 6 months, sought healthcare for diabetes or hypertension or other cardiovascular disease in a health facility	Yes	114 (80.3)	93 (66.9)	0.011
	No	28 (19.7)	46 (33.1)	
Were all the medications you needed available at the facility you sought care from?	Yes	59 (51.8)	43 (46.2)	0.430
	No	55 (48.3)	50 (53.8)	
What was/were the reasons why you were unable to obtain the medicines from the health facility you visited?	Too expensive	4 (7.3)	17 (34.0)	0.008
	Medicine(s) not in stock	50 (90.9)	43 (86.0)	
	Referred elsewhere by the medical professional	9 (16.4)	8 (16.0)	
	Other	2 (3.6)	-	
	Don’t know/Refused	-	-	
**Blood pressure**				
Mean systolic blood pressure (for HT only group)		154.4 (19.4)	161.1 (19.3)	0.0226
Mean diastolic blood pressure (for HT only group)		99.6 (11.3)	100.0 (11.7)	0.812
**Anthropometry**				
BMI (mean, SD)		28.1 (28.8)	33.6 (40.4)	0.109
Waist circumference (mean, SD)		95.3 (13.7)	98.8 (15.6)	0.044
Hip circumference (mean, SD)		105.5 (14.1)	104.9 (14.8)	0.742
Waist–hip ratio		0.91 (0.1)	0.95 (0.1)	<0.001
**Glycaemic parameters**				
Fasting blood glucose (mean, SD)		12.4 (5.2)	11.4 (4.7)	0.260
HbA1C (mean, SD)		10.9 (2.8)	10.5 (3.0)	0.524

SD, Standard deviation; KES, Kenya shillings; BMI, Body Mass Index; HbA1C, Glycated haemoglobin.

*Chi-square tests for categorical variables, and T-tests of association for continuous variables.

Both the intervention and control groups had similar dietary habits in terms of fruit and vegetable consumption, as well as comparable rates of current smoking. However, there were notable differences between the two groups. Participants in the intervention arm consumed more salt, while those in the control arm had a higher intake of sugar. Additionally, individuals in the control group were more physically active compared to those in the intervention group.

### Impact of the intervention on outcomes

The impact of the intervention is shown in Table 3. In both study arms, we observed reductions in HbA1c, blood pressure, and the proportion of participants with uncontrolled diabetes or hypertension after 6 months of follow-up. There was a reduction in mean HbA1c by 1.7% and 0.9% in the intervention and control arms, respectively. For blood pressure, there was a reduction in systolic BP by 6.3 and 7.6 mmHg in the intervention and control arms, respectively. From the difference in difference analysis (Table 4), there was a significantly greater reduction in HbA1c in the intervention arm than in the control arm. No major changes were observed in other comes except for a reduction in waist–hip ratio in the control arm (Table 3).

**Table 3.** Impact of the self-financing patient support group care model on mean blood pressure, glycated haemoglobin, body mass index, and waist–hip ratio among individuals with uncontrolled hypertension or diabetes in informal settlements in Nairobi, Kenya.

		Intervention (Viwandani)				Control (Korogocho)			
Variable		Baseline	Endline*	Difference	*P*-value**	Baseline	Endline*	Difference	*P*-value**
Glycated haemoglobin	*N*	45	37			48	44		
	Mean (95% CI)	10.8	9.0	−1.7 (−2.4, −0.9)	<0.001	10.6	9.9	−0.9 (−1.5, −0.3)	0.005
Uncontrolled diabetes	*N* (%)	45 (100%)	36 (97%)		0.013	48 (100%)	42 (95%)		0.221
Systolic blood pressure	*N*	62	62			75	75		
	Mean (95% CI)	155.0	148.7	−6.3 (−11.7, −0.9)	0.022	160.1	152.5	−7.6 (−12.9, −2.3)	0.005
Diastolic blood pressure	*N*	62	62			75	75		
	Mean (95% CI)	99.1	97.9	−1.1 (−4.2, 1.9)	0.462	99.7	94.8	−4.9 (−7.8, −2.0)	0.001
Uncontrolled hypertension	*N* (%)	62 (100%)	51 (82%)		0.003	75 (100%)	59 (79%)		<0.001
Body mass index	*N*	103	107			117	123	117	
	Mean (95% CI)	28.1	28.2	0.1 (−0.5, 0.7)	0.793	34.4	27.9	−6.5 (−14.6, 1.6)	0.113
Waist–hip ratio	*N*	103	107			117	123		
	Mean (95% CI)	0.91	0.92	0.01 (−0.01, 0.03)	0.257	0.95	0.90	−0.04 (−0.06, −0.02)	<0.001

CI, confidence interval; N, number of participants analysed; uncontrolled diabetes, HbA1C ≥ 7%; uncontrolled hypertension, SBP ≥ 140 and/or DBP ≥ 90 mmHg at endline.

*Endline survey conducted after 6 months of the intervention.

**Binomial proportion tests and chi-square tests of association.

**Table 4.** Difference in difference models for changes in blood pressure and glycaemic parameters (adjusted for age, sex, body mass index, and exercise).

	Systolic BP	Diastolic BP	HbA1c
Intercept	146.993***	116.236***	15.958***
	(8.085)	(4.702)	(1.494)
Sex (ref = female)	−1.592	−0.515	−0.100
Male	(3.474)	(2.025)	(0.588)
Age (years)	0.244+	−0.303***	−0.057**
	(0.145)	(0.085)	(0.019)
Physical activity	0.628	0.293	−0.084
	(0.466)	(0.263)	(0.059)
BMI	0.030	0.003	−0.088*
	(0.040)	(0.023)	(0.039)
Study arm (ref = control)	−5.185	−2.730	0.078
Intervention	(3.507)	(2.019)	(0.490)
Survey timepoints (baseline, endline)	−9.053***	−5.723***	−0.891**
	(2.673)	(1.484)	(0.317)
Intervention × survey timepoints	1.911	4.085+	−0.942*
	(3.933)	(2.186)	(0.463)
Standard deviation of the random effect for individuals	14.052	8.497	2.139
Standard deviation of the error term	15.990	8.849	1.489

HbA1c, glycated haemoglobin.

+*p* < 0.1; *p < 0.05; **p < 0.01; ***p < 0.001.

### Intervention uptake, feasibility, and experience from the roll-out

In the intervention arm, 60% (36/60) of the eligible participants with diabetes and 51% (44/87) of those with hypertension took up the intervention (joined and participated in the self-financing patient support groups). The experience with various components of the intervention was as follows.

#### Reach out activities

Participants were mobilised by a collaborative effort involving the research team, health facility team, sub-county non-communicable diseases office, and community health volunteers. The channels through which participants became aware of the intervention varied, with word of mouth from community health volunteers being the most prevalent at 42%. Following closely behind was the research team at 31%, while healthcare providers and other patients contributed 12% and 10%, respectively.

#### Group creation and membership

Mobilisation of the patients involved a series of preparatory meetings. The intervention was explained to them at the inception meeting, and any questions answered. Participants were split into three groups: one for diabetes (50 members) and two for hypertension (44 and 43 members). The subsequent meetings were at group level to elect leaders, set rules, and issue tools and devices. A representative from the health facility (Lungalunga Health Centre) was co-opted as a member of the support groups. The sub-county NCD office assisted in training the group leaders. Mobilisation and creation of the groups took 2 months (December 2022–January 2023). Reasons for individuals opting not to join groups included disinterest, lack of trust, tight work schedules, gaps in understanding the advantages of patient support groups, financial constraints preventing contributions, absence of financial incentives for group members, receiving sufficient medication directly from the healthcare facilities, and a lack of knowledge regarding the process of joining such groups.

#### Group function

All groups set rules for managing the functioning of the group. These included rules on membership, leadership composition, regulation of meetings, membership contributions, and financial management. During the intervention period, the challenges reported included non-compliance with contributions, low meeting attendance, lack of medication, inadequate knowledge of patient support groups, non-adherence to health education and advice, and poor health-seeking behaviour.

#### Group leadership

Each group elected, by voting, a chairperson, secretary, and treasurer.

#### Meetings

The groups held monthly meetings at the health facility. Members received reminders mainly by phone calls (50%), text messages (43%), and in-person (7%). These reminders came from the group leaders, community health volunteers, and the research team. Fifty-four percent of the group members attended all meetings during the intervention period. Reasons for non-attendance included being unwell, lack of transport, work, having travelled elsewhere on the day of the meeting, and being unaware.

#### Tool provision to the group

Blood pressure machines, glucometers and testing strips, and registers/recording tools were provided by the research team.

#### Self-care

During meetings, measurements of blood pressure and blood glucose were taken by the group members and recorded. Seventy-eight percent of the patients with diabetes reported having tested their blood sugar during the meetings. These tests were performed by a healthcare worker. Eighty-eight percent (88%) of the group members reported having their blood pressure measurements taken during the meetings. These measurements were mainly done by fellow group members (70%). The groups also received health education from staff at the health facility.

#### Financial management

All groups agreed to make financial contributions to cover registration fees and monthly membership fees. The average monthly contribution in Kenya shillings was 181.9 (USD 1.5), and this contribution was to cover medication costs. Seventy-one percent reported times when they were unable to pay the contribution. Reasons for not contributing included lack of money and mistrust.

#### Economic empowerment

The groups were not intended to be saving credit schemes. At the endline survey, no group had initiated discussions on becoming one.

#### Health facility support

All the groups were linked to Lungalunga Health Center. The healthcare workers helped in group initiation and support. Support received from the facility included health education, counselling, provision of meeting rooms, consultations, tools for BP and blood sugar measurement, and storage and dispensing of medications.

#### Community health team support

The community health volunteers supported the groups in mobilisation to join meetings/groups, providing reminders and health education.

#### Local government support

The sub-county NCD office assisted with mobilisation and group formation. The office also provided health talks to the groups during meetings and supported the groups with registration.

#### Other (external support to groups)

Ten percent (10%) reported receiving external support in form of tools, health education, group registration, and medications.

Some challenges were experienced during the roll-out. Formation of groups took longer than anticipated. Other organisations had similar patient support group activities in the same community. Also, there were existing patient support groups in the same health facility. Concerning the financial contributions, the groups set very low amounts and payment compliance was poor. Turn-up for group meetings was modest and some group leaders were not very active in running group activities. Overall, deploying the intervention was logistically and operationally feasible.

## Discussion

In this study, we have shown that a co-created self-financing patient support group care model is feasible and can impact disease outcomes in a low-resource setting. The intervention resulted in a significantly greater improvement (reduction) in mean HbA1c in patients with uncontrolled diabetes compared to the control group although there was a similar degree of blood pressure reduction in the two groups. There was also no impact on the proportion of participants with uncontrolled hypertension or diabetes.

The improvement in HbA1c in the intervention arm is in agreement with other self-financing patient support groups have been shown to improve glycaemic parameters (HbA1c and fasting blood glucose) in patients with diabetes [17]. Also, the concept of patient support groups embodies a significant ideological shift, moving away from the traditional notion of patients as passive recipients of treatment to individuals empowered to actively engage as partners in their health management (21). These support groups, characterised by peer interaction and shared experiences, are beneficial for individuals with hypertension and/or diabetes [22]. In fostering a sense of community and shared responsibility, these peer groups play a pivotal role in empowering patients, promoting knowledge exchange, and cultivating a supportive environment conducive to effective health management.

The intervention had no impact on mean blood pressure, contrary to reports from other studies elsewhere. For example, initiatives in rural Kenya [23] and Cambodia [24] demonstrated significant declines in blood pressure, showcasing alternative successful approaches in resource-limited settings. The lack of impact of the intervention could be attributed to the relatively low uptake of the intervention. Only half of the participants with hypertension in the intervention arm took up the intervention. A higher uptake may have yielded different results. In addition, the duration of the intervention (6 months) may have been too short. Perhaps, the groups needed more time to accumulate enough contributions for the medications, considering that they were in the study for only 6 months and had few members in the groups. To achieve economies of scale, there is a need to have more time to save and enough members to accumulate money over time. We also do not know the availability of medicines at the healthcare facilities. Could it be that diabetes medications were more available during the study period? Our intervention anchors on topping up medicines in the facility and the supply of medicines in the facility could influence. Trials with longer follow-up durations, preferably over 1 year, are recommended. For, example, a cluster-randomised trial conducted in Kenya showed that an intervention with group medical visits and microfinance for patients with diabetes or hypertension resulted in substantial reductions in systolic blood pressure over 1 year [25]. It is also important to note that there were substantial improvements in the parameters measured among participants in the control arm. These improvements could have dampened the impact of the intervention.

Despite the intervention having no impact on the proportion of patients with uncontrolled diabetes or hypertension, there were improvements in HbA1c and blood pressure parameters in both study arms. The improvements in the control arm could be attributed to the standard care received, regression to the mean or placebo effect. However, the improvements noted in both arms are clinically significant. Improvement in HbA1c or blood pressure, even without surpassing the set cut-off for control, substantially reduces the risk of complications from the disease. For example, a unit reduction in Hba1c has considerable impact on microvascular complications [26–28]. Also, a reduction in systolic blood pressure of 5 mmHg reduces the risk of developing major cardiovascular events by 10% [29].

Considering the limited evidence on self-financing patient support groups for diabetes and hypertension, it was reassuring to demonstrate feasibility of the intervention. Important lessons were learnt. The trial recruited known patients with uncontrolled diabetes and hypertension, who are not members of existing patient support groups but live in a community which has existing support groups for diabetes and hypertension. An intervention also targeting newly diagnosed patients may have a higher uptake. When conducting similar studies in the future, a longer period should be set for participant mobilisation and group creation. Before rolling out such interventions, mapping of stakeholders (non-governmental organisations, government programmes, and researchers) doing similar work in the set study areas, is important. Another potential hiccup is the low financial contributions set by members. Careful considerations should be made on the most appropriate target population, requirement for seed funding, and a more objective approach to setting member contributions.

The strength of our study lies in its novelty, particularly in introducing the unique self-financing patient support group care model. Also, co-design of the intervention with stakeholders is a strength. However, some limitations must be acknowledged. For example, the study does not isolate the distinct benefits derived from self-financing as opposed to those originating from participation in a patient support group. We did not measure financial outcomes and medicine access in the health facilities, cost of the intervention in the research and non-research setting. These issues can affect the group success and control of clinical parameters. We did not measure the impact on social cohesion and trust, which are important elements of such an intervention.

## Conclusions

A self-financing patient support group care model is feasible. The model provides an additional toolkit for the management of hypertension and diabetes in resource-limited settings and can be adapted for other chronic diseases. It is imperative that more evidence on the effectiveness of this care model is obtained through larger trials and in diverse settings to confirm findings, examine whether effects are maintained and assess implementation outcomes.

## Data Availability

The datasets used and/or analysed during the current study are available from the corresponding author, Richard Sanya, on reasonable request.
